# Growth Differentiation Factor 11 Promotes Neurovascular Recovery After Stroke in Mice

**DOI:** 10.3389/fncel.2018.00205

**Published:** 2018-07-16

**Authors:** Lu Lu, Xiaofei Bai, Yongliang Cao, Haiyu Luo, Xing Yang, Lijing Kang, Mei-Juan Shi, Wenying Fan, Bing-Qiao Zhao

**Affiliations:** Department of Translational Neuroscience, Jing’an District Centre Hospital of Shanghai, State Key Laboratory of Medical Neurobiology and Institutes of Brain Science, Fudan University, Shanghai, China

**Keywords:** stroke, neurogenesis, angiogenesis, functional recovery, GDF11

## Abstract

**Background:** Growth differentiation factor 11 (GDF11), a member of transforming growth factor-β (TGF-β) superfamily, was shown to rejuvenate cardiac and skeletal muscle function and to improve cerebral vasculature and neurogenesis in old mice. However, recent experimental data reported that raising GDF11 levels inhibited skeletal muscle regeneration and had no effect on cardiac hypertrophy. Our aim was to investigate the effects of GDF11 on brain repair during the recovery phase after stroke.

**Methods:** Mice were subjected to distal middle cerebral artery occlusion, and recombinant GDF11 (rGDF11) was injected intraperitoneally once a day during days 7–13 after stroke. Neuronal precursor cells (NPCs) proliferation and angiogenesis were assayed at 14 days. Neuronal regeneration was assayed at 42 days. The beam-walking test and CatWalk were used to evaluate behavioral functions. Downstream pathways of GDF11 were also investigated.

**Results:** GDF11 was upregulated in the ipsilateral peri-infarct cortex and subventricular zone (SVZ) at 14 days after stroke. Treatment with rGDF11 enhanced the number of newborn NPCs and endothelial cells, microvascular length and area, and brain capillary perfusion. Western blots showed that rGDF11 upregulated brain-derived neurotrophic factor (BDNF) and increased the levels of proangiogenic factor angiopoietin-2 (Ang-2) and phosphorylation of vascular endothelial growth factor receptor-2 (VEGFR-2). We also found that rGDF11 upregulated the transcription factors Smad2 and Smad3 phosphorylation, but these activations were blocked by a TGF-β receptor inhibitor SB431542. Moreover, rGDF11-induced angiogenic remodeling and NPCs proliferation were reversed by injection of SB431542, suggesting that GDF11 may exert its effect via the TGF-β/Smad2/3 signaling pathway. Finally, treating mice with rGDF11 resulted in a significant increase in neuronal regeneration and functional recovery.

**Conclusion:** GDF11 promoted neurogenesis and angiogenesis and contributed to functional recovery after stroke in mice.

## Introduction

Stroke is the leading cause of disability around the world (Murray et al., 2012). However, currently there is no effective treatment to facilitate the recovery in stroke patients. Stroke triggers the proliferation of the neural progenitor cells (NPCs) in the subventricular zone (SVZ) and the subgranular zone (SGZ) and the migration of NPCs toward the stroke areas (Arvidsson et al., 2002; Teng et al., 2008; Osman et al., 2011). Recent studies have suggested that stroke also induces angiogenesis in the peri-infarct region (Jiang et al., 2016). In stroke patients, there is a significant correlation between the vessel density in the brain and delayed mortality, suggesting that the angiogenesis is important for stroke recovery (Krupinski et al., 1993; Krupinski, 1994). Furthermore, angiogenic vessels were reported to release growth factors and chemokines to promote the migration of neuroblasts and the survival of new neurons, indicating that angiogenesis is highly linked with neurogenesis (Tsai et al., 2006). Therefore, therapeutic approaches to promote both neurogenesis and angiogenesis process may provide promising opportunities for stroke recovery.

Growth differentiation factor 11 (GDF11), a member of the transforming growth factor-β (TGF-β) superfamily, participates various biological processes in mammals. GDF11 has been identified as a rejuvenation factor which could reverse age-related cardiac hypertrophy and improve muscle and brain function (Loffredo et al., 2013; Katsimpardi et al., 2014; Sinha et al., 2014). The major findings of these studies were that circulating levels of GDF11 decreased with aging and recombinant GDF11 injection could improve the vascular remodeling and increase neurogenesis in aging mice (Katsimpardi et al., 2014). However, a recent report questioned the conclusion and suggested that circulating GDF11 levels increased with age and reduced muscle regeneration (Egerman et al., 2015). Another study also demonstrated a negative effect of GDF11 on age-related cardiac hypertrophy (Smith et al., 2015). Furthermore, *in vitro* experiments found that GDF11 treatment could increase the peripheral blood endothelial progenitor cells migration and the sprout formation (Finkenzeller et al., 2015), while showed no significant effect on the human umbilical vein endothelial cells proliferation and migration (Zhang et al., 2016).

In this study, we investigated the role of GDF11 on stroke recovery in a mouse model of distal occlusion of middle cerebral artery. We found that delayed treatment with recombinant GDF11 (rGDF11) at 7 days after stroke promoted neurogenesis and angiogenesis and improved behavioral outcome by regulating the TGF-β/Smad2/3 signaling pathway.

## Materials and Methods

### Distal Middle Cerebral Artery Occlusion

Adult (8–10 weeks old) male C57BL/6 mice were purchased from Shanghai SLAC Laboratory Animal Co., Ltd. (Shanghai, China). All experimental procedures were approved by the Animal Care and Use Committee of Institutes of Brain Science, Fudan University. The procedure of dMCAO was performed as described previously (Xu et al., 2017). Mice were anesthetized with isoflurane via facemask (3% for induction, 1–1.5% for maintenance in 70% nitrous oxide and 30% oxygen). The skin was incised between the right eye and the right ear, the temporal muscle was removed to perform the craniotomy. After resecting the dura mater, the distal portion of the MCA was exposed and electrocoagulated permanently. Then the right common carotid artery (CCA) was ligated for 15 min immediately after the MCA occlusion. Body temperature was maintained at 37 ± 0.5°C throughout the surgery with a heating board. The sham surgery only exposed the MCA and CCA but not to perform the coagulation or the ligation. Treatment groups were assigned in a randomized and blinded manner.

### Drugs and Treatments

To test the efficacy of delayed injection of rGDF11 after stroke, mice were given a daily intraperitoneal injection of human recombinant GDF11 (# 120-11, PeproTech, Rocky Hill, NJ, United States) at 0.1 mg/kg or vehicle (saline) during days 7–13 after stroke (Katsimpardi et al., 2014). For the *in vivo* inhibition of the TGF-β signaling, intraperitoneal injections of SB431542 (10 mg/kg, Selleck Chemicals, Houston, TX, United States) or vehicle control (50% [v/v] DMSO in sterile saline) were given once a day during days 7–13 (Inman et al., 2002; Zhao et al., 2015). To label the newborn cells, bromodeoxyuridine (BrdU; 50 mg/kg, Sigma-Aldrich, St. Louis, MO, United States) was given intraperitoneally twice a day during days 8–13 after stroke. Mice were killed at 14 or 42 days.

### *In Vivo* Tomato-Lectin Angiography

Mice received transcardially injections of biotinylated lycopersicon esculentum (tomato) lectin (tomato-lectin; 1.25 mg/kg, B-1175, Vector Laboratories, Burlingame, CA, United States) 5 min prior to sacrifice at 14 days after stroke (Xu et al., 2017). Brain cryosections (20 μm) were immunostained with fluorescence-streptavidin (Vector Laboratories) to label endothelial cells only in perfused vessels.

### Immunofluorescence

At 14 days after stroke, mice were perfused with ice-cold PBS followed by 4% phosphate-buffered paraformaldehyde. Brains were removed and cryoprotected in 30% sucrose at 4°C overnight. Frozen coronal sections (20 μm thick) were cut using a cryostat (CM1950 Leica Microsystems Inc., Buffalo Grove, IL, United States) and stored at -80°C. Immunohistochemistry was performed as described previously (Zhao et al., 2004). The primary antibodies were: goat anti-doublecortin (DCX, Santa Cruz Biotechnology, Santa Cruz, CA, United States), rat anti-BrdU (ab6326, Abcam, Cambridge, MA), rabbit anti-Sox2 (ab92494, Abcam), goat anti-CD31 (AF3628, R&D Systems, Minneapolis, MN, United States), mouse anti-NeuN (MAB377, Millipore, MA, United States). The secondary antibodies used were: Alexa Fluor 594-conjugated donkey anti-rabbit IgG, Alexa Fluor 594-conjugated donkey anti-goat IgG, Alexa Fluor 594-conjugated donkey anti-mouse IgG, Alexa Fluor 488-conjugated donkey anti-rat IgG (all from Invitrogen, Waltham, MA, United States).

### Image Analysis and Quantification

Three serial sections per animal spaced 600 μm apart (-0.1 to 1.10 mm from the bregma) were prepared for immunohistochemical quantifications. Images were captured with an Olympus BX 51 microscope and Olympus FV1000 confocal microscope and analyzed with ImageJ software. For quantification of BrdU^+^, Sox2^+^, DCX^+^/BrdU^+^, and Sox2^+^/BrdU^+^ cells, 5 fields of view from the SVZ were digitalized under a ×40 objective lens using an epifluorescence microscope. For NeuN^+^/BrdU^+^ immunostaining, three fields of view from the peri-infarct cortical areas were digitalized using a ×40 objective lens. Each image was traced using ImageJ software. The total numbers of positive cells in the traced area were counted. Data are expressed as the number of positive cells per mm^2^. For quantification of angiogenesis, three fields of view from the peri-infarct cortical areas were digitalized using a ×40 objective lens. The length and area of CD31-positive vessels and the number of vessel branch points were measured using the Image J analysis tool. For lectin-perfused vessels, the thresholded CD31 and tomato-lectin images were superimposed in two different layers. The CD31-positive area was selected, and the mean value of the pixels of the tomato-lectin image was calculated within the CD31 selection. Lectin-perfused vessels were presented as a percentage of tomato-lectin-positive area covering CD31-positive area. The total numbers of BrdU-positive cells in the CD31-positive vessels were counted and expressed as the number of CD31^+^/BrdU^+^ per mm^2^. Five to eight mice were used in each group.

### Western Blot

At 14 days after stroke, mice were killed by overdose of chloral hydrate. Ischemic brain tissues and matching tissue from sham-operated mice were dissected, and the levels of intrinsic GDF11 expressed by the animal were examined. Western blot was performed as reported previously (Fan et al., 2014). The primary antibodies were: rabbit anti-GDF11 (ab124721, Abcam), rabbit anti-Ang-2 (ab8452, Abcam), rabbit anti-BDNF (ab108319, Abcam), mouse anti-thrombospondin-1 (TSP-1; clone Ab-11; Lab Vision, Fremont, CA, United States), rabbit anti-VEGFR-2 (2479, Cell Signaling Technology, Beverly, MA, United States), rabbit anti-phospho-VEGFR-2 (pVEGFR-2; ab76466, Abcam), rabbit anti-Smad2/3 (3102, Cell Signaling Technology), rabbit anti-p-Smad2 (3108, Cell Signaling Technology), rabbit anti-p-Smad3 (9520, Cell Signaling Technology), rabbit anti-β-actin (4970, Cell Signaling Technology), rabbit anti-GAPDH (5174, Cell Signaling Technology). The secondary antibodies included anti-rabbit (7074, Cell Signaling Technology) or anti-mouse (7076, Cell Signaling Technology) HRP-conjugated antibodies.

### Behavior Tests

To assess functional outcomes, beam walking and gait analysis were used to evaluate the motor and sensorimotor asymmetries, respectively. The beam walking test were performed blindly before surgery and were repeated at 1, 7, 14, 28, and 42 days after stroke as described previously (Fan et al., 2014). Gait analysis was performed with an automated computer-assisted method (CatWalk^TM^, Noldus Information Technology, Wageningen, The Netherlands) in accordance with the manufacturer’s instructions and published procedures (Balkaya et al., 2013; Caballerogarrido et al., 2015). In brief, mice were allowed to move freely across an elevated 1.3 m long glass platform. Footprints were captured by a high-speed camera and analyzed on the computer using the Catwalk XT 8.1 software. The animals were trained for 3 days before surgery and tested at days 1, 7, 14, 28, 42 after stroke. The max contact area and the print length were used to evaluate the influence of the rGDF11 treatment to the mice after stroke.

### Statistical Analysis

Data are reported as mean ± SD. One-way analysis of variance (ANOVA) was used to detect statistical significance followed by the Boneferroni’s multiple comparison test with GraphPad Prism 6. When 2 groups were compared, unpaired 2-tailed Student *t*-test was used. Statistical significance was assigned for *P* < 0.05.

## Results

### rGDF11 Enhanced NPCs Proliferation and Angiogenesis After Stroke

**FIGURE 1:**
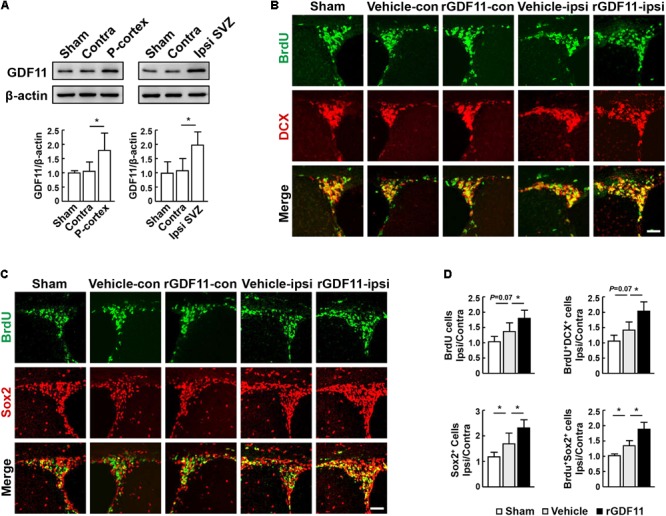
Treatment with rGDF11 enhanced neuronal precursor cells (NPCs) proliferation in ischemic mice. **(A)** Representative immunoblots and quantification of GDF11 in the sham-operated mice, the contralateral (Contra), ipsilateral (Ipsi) peri-infarct cortex (P-cortex), and subventricular zone (Ipsi SVZ) of mice at 14 days after stroke. *n* = 5 per group. **(B,C)** Double immunostaining of BrdU with DCX **(B)** and Sox2 **(C)** in the SVZ from sham-operated mice, the contralateral (con) and ipsilateral (ipsi) SVZ from the vehicle and rGDF11 treated mice at 14 days after stroke. Scale bar = 40 μm. **(D)** Quantitative determination of BrdU^+^, BrdU^+^/DCX^+^, Sox2^+^, and BrdU^+^/Sox2^+^ cells for each group. The data are presented as a ratio of ipsilateral/contralateral cell count. *n* = 6 per group. Values are mean ± SD. ^∗^*P* < 0.05.

Western blot analysis showed that GDF11 was significantly upregulated in the ipsilateral peri-infarct cortex and SVZ compared to the contralateral hemisphere and the sham-operated mice at 14 days after stroke (**Figure 1A**). Treatment with rGDF11 at 7 days after stroke resulted in a significant increase in the ipsilateral/contralateral ratio of BrdU^+^ cells, DCX^+^/BrdU^+^ neuroblasts (**Figures 1B,D**), Sox2^+^ NPCs and Sox2^+^/BrdU^+^ cells (**Figures 1C,D**) in SVZ compared with the vehicle-treated mice, indicating that rGDF11 treatment promoted NPCs proliferation after stroke.

**FIGURE 2:**
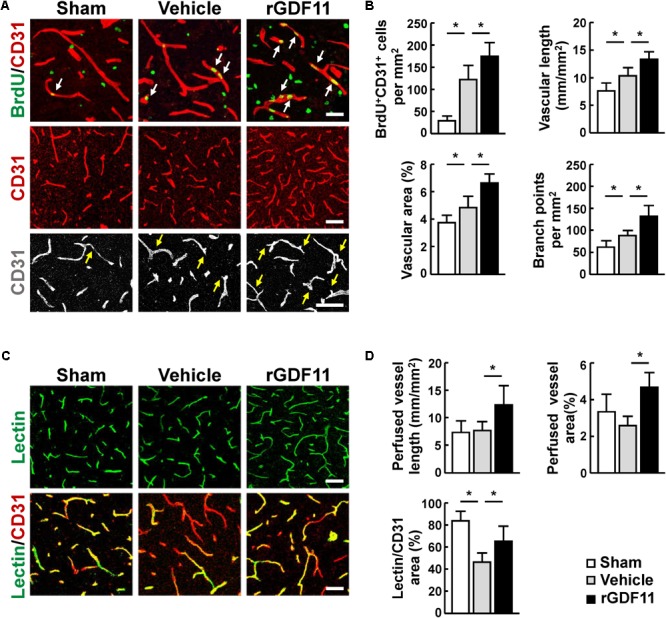
Treatment with rGDF11 promoted neovascularization in ischemic mice. **(A)** Representative images of BrdU^+^/CD31^+^ endothelial cells, CD31^+^ microvessels, and vascular branches in the peri-infarct cortical areas from sham-operated mice and mice treated with vehicle or rGDF11 at 14 days after stroke. Scale bar = 40 μm. **(B)** Quantification of BrdU^+^/CD31^+^ endothelial cells, CD31-positive microvascular length, percent area occupied by CD31-positive vascular structures, and vascular branches for each group. *n* = 6–8 per group. **(C)** At 14 days after stroke, mice were given an intravascular injection of tomato-lectin, and brain sections were stained with or without CD31. Shown are representative images from sham-operated mice and mice treated with vehicle or rGDF11. Scale bar = 40 μm (upper panel) and 60 μm (lower panel). **(D)** Quantification of lectin-perfused vessel length, percent area occupied by lectin-perfused vessels, and tomato-lectin and CD31 double-labeled vessels for each group. *n* = 5 per group. Values are mean ± SD. ^∗^*P* < 0.05.

**FIGURE 3:**
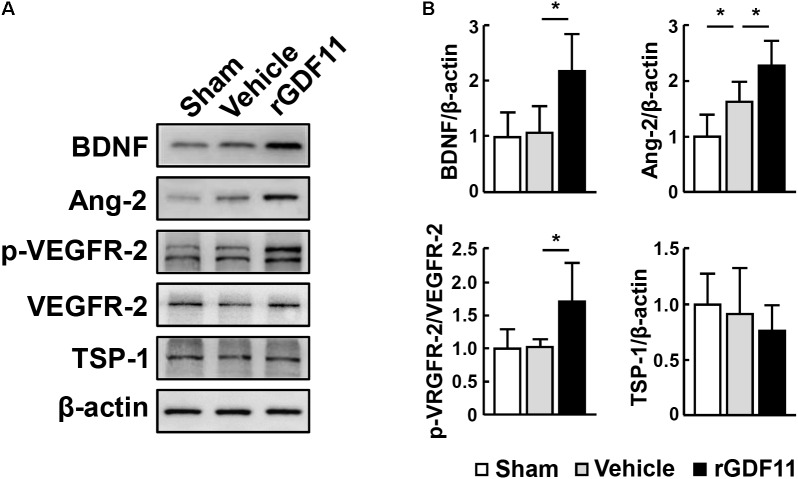
rGDF11 upregulated neurotrophic and proangiogenic factors. **(A)** Representative immunoblots of BDNF, Ang-2, p-VEGFR-2, VEGFR-2, and TSP-1 from sham-operated mice and mice treated with vehicle or rGDF11 at 14 days after stroke. **(B)** Quantification of BDNF, Ang-2, p-VEGFR-2, and TSP-1 for each group. *n* = 5 per group. Values are mean ± SD. ^∗^*P* < 0.05.

We then investigated the effect of rGDF11 on angiogenesis at 14 days after stroke and found that rGDF11-treated mice showed a significant increase in CD31-positive microvascular length and area and the number of vascular branches. The amount of newborn vascular endothelia cells labeled with BrdU^+^/CD31^+^ was also markedly increased in the rGDF11-treated mice (**Figures 2A,B**). Moreover, treatment with rGDF11 significantly increased the lectin-perfused vessel length and area in the peri-infarct cortical areas, and the colocalized area of tomato-lectin and CD31 positive vessels compared to the vehicle group (**Figures 2C,D**). Western blot assays of brain homogenates from the peri-infarct cortex indicated that rGDF11 significantly upregulated the neurotrophic factor BDNF (**Figures 3A,B**). We also found that the levels of proangiogenic factors Ang-2 and VEGFR-2 phosphorylation were upregulated in the peri-infarct cortex of rGDF11-treated mice, although no significant change of thrombospondin-1 was found. Together, these data indicated that rGDF11 increased NPCs proliferation and angiogenesis, which may play a crucial role during the stroke recovery.

### TGF-β-Smads Signaling Mediated the Effects of rGDF11 in Promoting NPCs Proliferation and Angiogenesis

**FIGURE 4:**
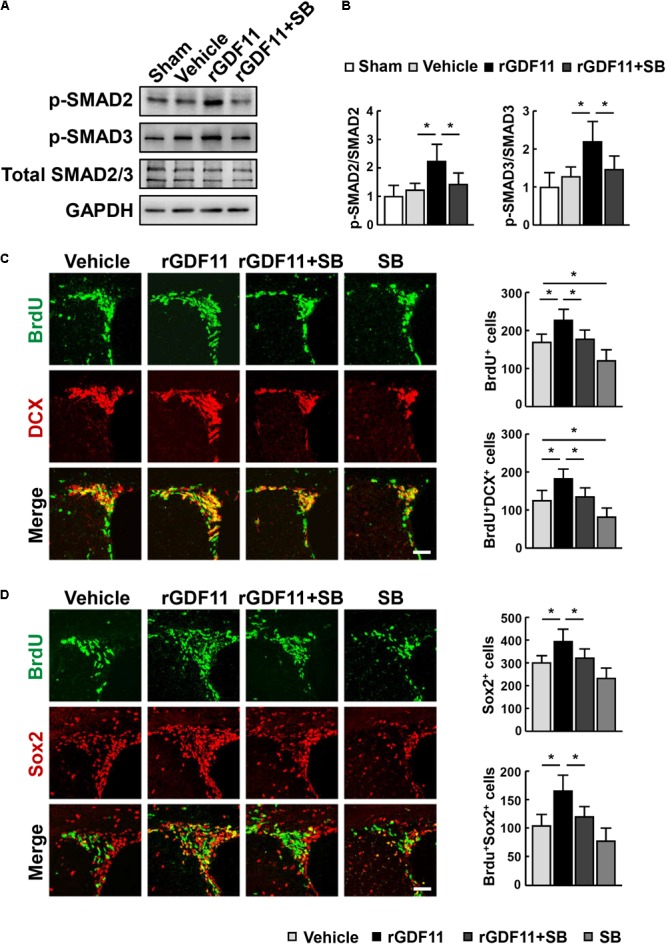
rGDF11 enhanced NPCs proliferation via TGF-β-Smads signaling. **(A)** Representative immunoblots of p-Smad2, p-Smad3, and total Smad2/3 from sham-operated mice and mice treated with vehicle, rGDF11 or rGDF11 in combination with SB431542 (SB) at 14 days after stroke. **(B)** Quantification of p-Smad2 and p-Smad3 for each group. *n* = 5 per group. **(C,D)** Double immunostaining of BrdU with DCX **(C)** and Sox2 **(D)** in the SVZ of mice treated with vehicle, rGDF11, rGDF11 in combination with SB431542 (SB) or SB431542, and quantitative determination of BrdU^+^, BrdU^+^/DCX^+^ **(C)**, Sox2^+^, and BrdU^+^/Sox2^+^ **(D)** cells for each group. Scale bar = 40 μm. *n* = 6 per group. Values are mean ± SD. ^∗^*P* < 0.05.

**FIGURE 5:**
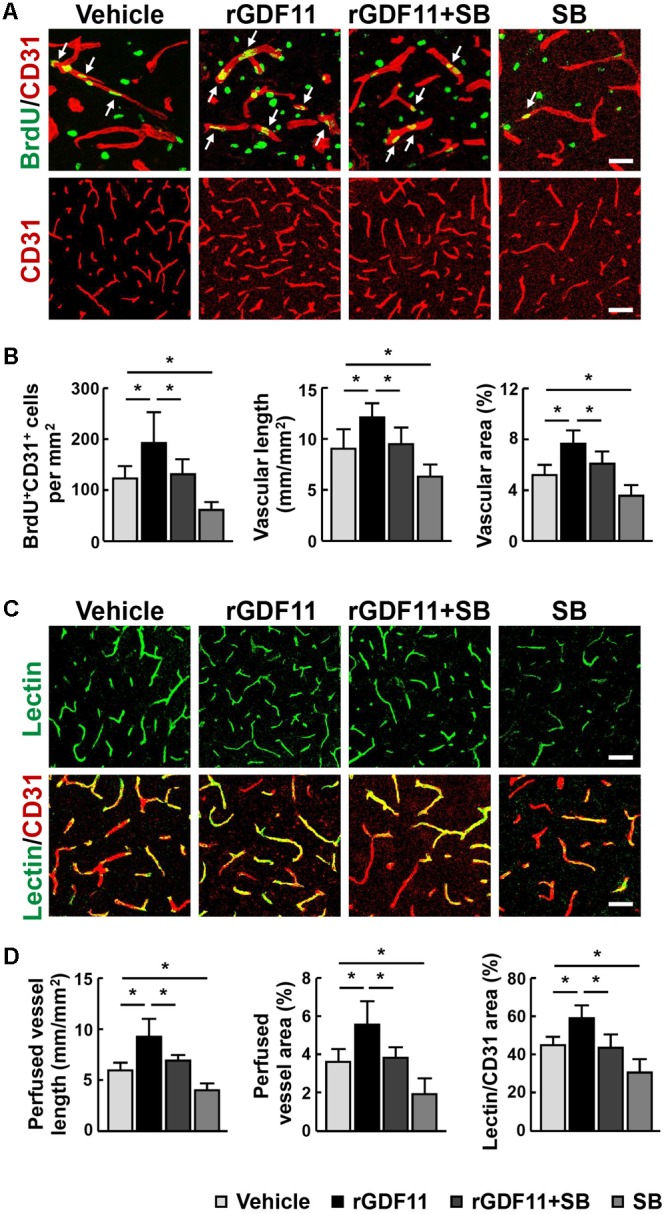
Inhibition of TGF-β reversed rGDF11-induced vascular remodeling. **(A)** Representative images of BrdU^+^/CD31^+^ endothelial cells and CD31^+^ microvessels in the peri-infarct cortical areas in mice treated with vehicle, rGDF11, rGDF11 in combination with SB431542 (SB), or SB431542 at 14 days after stroke. Scale bar = 40 μm (upper panel) and 60 μm (lower panel). **(B)** Quantification of BrdU^+^/CD31^+^ endothelial cells, CD31-positive microvascular length, and percent area occupied by CD31-positive vascular structures for each group. *n* = 6 per group. **(C)** Representative images of tomato-lectin perfused vessels and tomato-lectin and CD31 double-labeled vessels in the peri-infarct cortical areas in mice treated with vehicle, rGDF11, rGDF11 in combination with SB431542 (SB), or SB431542. Scale bar = 40 μm (upper panel) and 60 μm (lower panel). **(D)** Quantification of lectin-perfused vessel length, percent area occupied by lectin-perfused vessels, and tomato-lectin and CD31 double-labeled vessels for each group. *n* = 5 per group. Values are mean ± SD. ^∗^*P* < 0.05.

Transcription factors Smads have been considered downstream effectors of GDF11 (Andersson, 2006). We found that treatment of mice with rGDF11 upregulated both Smad2 and Smad3 phosphorylation in brain homogenates (**Figures 4A,B**). To test the hypothesis that rGDF11 may contribute to functional recovery after stroke by regulating the TGF-β/Smads signaling, we compared mice treated with the TGF-β type I receptor inhibitor SB431542 with control mice treated with vehicle. Western blot confirmed that SB431542 partially blocked the activation of Smad2 and Smad3 by rGDF11. As expected, SB431542 suppressed rGDF11 induced-increase in the number of BrdU^+^ cells, DCX^+^/BrdU^+^ neuroblasts, Sox2^+^ NPCs and Sox2^+^/BrdU^+^ cells in the SVZ (**Figures 4C,D**). SB431542 also led to a significant decrease in BrdU^+^/CD31^+^ endothelia cells, microvascular length and area, and perfused vessels in mice treated with rGDF11 (**Figures 5A–D**).

### rGDF11 Increased Neuronal Regeneration and Improved Functional Recovery After Stroke

**FIGURE 6:**
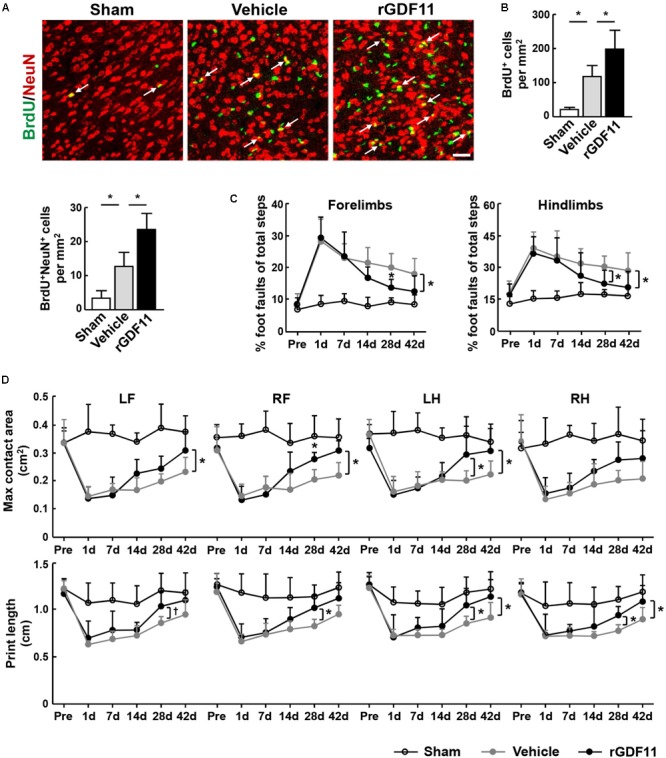
Treatment with rGDF11 promoted neuronal regeneration and improved functional recovery after stroke. **(A,B)** Representative images and quantitative determinations of BrdU-positive neurons (NeuN) in the peri-infarct cortical areas of sham-operated mice and mice treated with vehicle or rGDF11 at 42 days. Scale bar = 40 μm. *n* = 6 per group. **(C)** Effects of rGDF11 on locomotor deficit. Analysis of forelimb (left) and hindlimb (right) footfaults was performed at the indicated times after stroke. *n* = 8 per group. **(D)** Effects of rGDF11 on sensorimotor performance. CatWalk gait parameters were assayed at the indicated times after stroke. LF, left forelimb; RF, right forelimb; LH, left hindlimb; RH, right hindlimb. *n* = 8 per group. Values are mean ± SD. ^†^*P* = 0.07, ^∗^*P* < 0.05.

To determine whether rGDF11 affects NPCs differentiation, we treated mice with either vehicle or rGDF11 at 7–13 days and examined brains at 42 days after stroke. Our results indicated that rGDF11 significantly increased the number of BrdU^+^ cells and BrdU^+^/NeuN^+^ cells in the peri-infarct cortex at 42 days (**Figures 6A,B**). The number of BrdU^+^/GFAP^+^ cells were comparable between control and rGDF11-treated mice (data not shown). These data suggested that rGDF11 promoted regeneration of neuronal cells. Measurements of motor activity by the beam walking test and sensorimotor performance by the CatWalk gait indicated no differences in behavioral deficits in rGDF11 group before treatment compared with vehicle-treated group at 1–7 days after stroke (**Figures 6C,D**). However, rGDF11-treated mice had profound improvement in long-term motor activity and sensorimotor performance.

## Discussion

In this study, we explored the effects of GDF11 on brain repair during the recovery phase after stroke. We found that GDF11 was upregulated in the ipsilateral SVZ and the peri-infarct cortex at 14 days after stroke. We also demonstrated that treatment with rGDF11 from 7 days after stroke significantly enhanced neurogenesis and angiogenesis and improved functional outcome through regulating the TGF-β/Smad2/3 signaling pathway.

GDF11 was proved to have widespread expression and play unique properties in multiple systems (Mcpherron, 2010). Initial studies showed that the serum levels of GDF11 declined with age and exogenous rGDF11 treatment could reverse the age-related dysfunction in skeletal muscle, heart and brain through rejuvenating stem cells, suggesting that GDF11 is one of the anti-aging factors (Loffredo et al., 2013; Katsimpardi et al., 2014; Sinha et al., 2014). However, other studies indicated that GDF-11 level did not change or increased with age, and had some negative effects on muscles, hearts and bones (Liu et al., 2016; Zimmers et al., 2017). It should be noted that the level of GDF11 may be a predictive biomarker of heart attack, stroke, congestive heart failure, and overall mortality in patients (Hall, 2014). Here we found that the ipsilateral peri-infarct cortex and SVZ level of GDF11 increased after stroke. Importantly, exogenous rGDF11 treatment promoted the NPCs proliferation in SVZ and neural maturation in the peri-infarct cortex and increased cerebral neovascularization and vessel remodeling process. Moreover, we also found that rGDF11 treatment effectively improved functional outcome as assessed by beam walking and catwalk tests at different time points after stroke. Previous work suggests that angiogenesis and neurogenesis are decreased after stroke in aging patients and animals (Lähteenvuo and Rosenzweig, 2012; Koh and Park, 2017). Recently, rGDF11 has been shown to stimulate vascular remodeling and increase neurogenesis in aging mice (Katsimpardi et al., 2014). These results, together with our findings, raise the possibility that GDF11 might also contribute to neurovascular repair after stroke in aging patients.

Neurotrophic and pro-angiogenic factors were reported to be crucial for neurogenesis and angiogenesis in the peri-infarct region after stroke (Slevin et al., 2006; Stanne et al., 2016). Previous studies have shown that BDNF could be secreted by newly formed blood vessels to maintain neurogenesis (Louissaint et al., 2002; Kim et al., 2008), and Ang-2 and VEGFR-2 activation were reported to promote the formation of neovessels in the ischemic boundary zone (Reiss et al., 2007; Ruan et al., 2015). Our results showed that rGDF11 treatment upregulated the levels of BDNF, Ang-2 and VEGFR-2 phosphorylation, suggesting that rGDF11 may exert its effect through regulating these factors.

It is known that GDF11 binds to the TGF-β type I receptors to activate the Smad2/Smad3 pathway (Andersson, 2006). Recent study also confirmed that GDF11 has a direct biological effect on primary brain capillary endothelial cells through the Smad2/3 pathway (Katsimpardi et al., 2014). We found that rGDF11 treatment led to a significant increase in Smad2/Smad3 phosphorylation, and the rGDF11-induced neurogenesis and angiogenesis were partially reversed by a TGF-β receptor inhibitor SB431542. These data suggested that GDF11 may promote the neurogenesis and angiogenesis after stroke via the phosphorylated Smad2/3 pathway. However, there are several limitations to this study that warrant further investigation. First, the mechanisms that recruit GDF11 into the ischemic brain still needs to be clarified. It can be speculated that the increased GDF11 levels observed in the ipsilateral ischemic hemispheres might be associated with the activities of proprotein convertases (PC) 5/6 and bone morphogenetic protein-1/tolloid (BMP1/TLD)-like proteinases. This hypothesis is supported by previous reports that both PC 5/6 and BMP1/TLD-like proteinases regulate the processing and activation of GDF11 via cleavage its prodomain (Ge et al., 2005; Essalmani et al., 2008). Second, we showed that the effects of rGDF11 may be associated with the TGF-β-Smads signaling. However, multiple other rGDF11 substrates may also be involved in the rGDF11 effects. Future studies are needed to carefully examine how rGDF11 may modulate angiogenesis and neurogenesis after stroke. Third, the young mice were used in the present study. However, stroke in humans predominantly occurs in the aging population (Ramirezlassepas, 1998). Translating these experimental results to the human situation should be done with caution.

## Conclusion

Our study suggest that GDF11 is a key regulator of neurovascular regeneration after stroke and this result may provide a potential therapeutic treatment for improving the functional outcome after stroke.

## Author Contributions

LL, XB, YC, HL, XY, LK, and M-JS performed the experiments and analyzed the data. LL, WF, and B-QZ designed the study and wrote the manuscript. All authors read and approved the final manuscript.

## Conflict of Interest Statement

The authors declare that the research was conducted in the absence of any commercial or financial relationships that could be construed as a potential conflict of interest.
